# “*Shared experience makes this all possible*”: documenting the guiding principles of peer-led services for people released from prison

**DOI:** 10.1186/s12889-023-17524-4

**Published:** 2024-01-03

**Authors:** Heather Palis, Pam Young, Mo Korchinski, Shawn Wood, Jessica Xavier, Nelson Luk, Simrat Mahil, Sofia Bartlett, Helen Brown, Amy Salmon, Tonia Nicholls, Amanda Slaunwhite

**Affiliations:** 1 Department of Psychiatry, University of British Columbia, 255 Wesbrook Mall, Vancouver, BC V6T 2A1 Canada; 2 British Columbia Centre for Disease Control, 655 W 12th Ave, Vancouver, BC V5Z 4R4 Canada; 3 Unlocking the Gates Services Society, 22838 Lougheed Hwy. Unit 104, Maple Ridge, BC V2X 2V6 Canada; 4 School of Population and Public Health, University of British Columbia, 2206 East Mall, Vancouver, BC V6T 1Z3 Canada; 5 School of Nursing, University of British Columbia, 2211 Wesbrook Mall T201, Vancouver, BC V6T 2B5 Canada; 6 Centre for Health Evaluation and Outcome Sciences, St. Paul’s Hospital, 70-1081 Burrard Street, Vancouver, BC V6Z IY6 Canada; 7 BC Mental Health and Substance Use Services, 4949 Heather St, Vancouver, BC V5Z 3L7 Canada; 8 Department of Psychiatry, University of British Columbia, BC Centre for Disease Control, 655 W 12th Avenue, Vancouver, BC V5Z 4R4 Canada

**Keywords:** Peer-led services, Community reintegration, Prison

## Abstract

**Background:**

There is a growing body of evidence demonstrating the effectiveness of peer-led services in supporting community reintegration for people released from prison. This study aims to document the guiding principle of a peer-led service for people released from prison, from the perspective of peer mentors.

**Methods:**

Data were collected using focus groups (*N* = 10; 2 groups with 5 participants each) and one-on-one interviews (*N* = 5) including a total of 13 people, representing all UTGSS staff at the time of the study. An inductive thematic analysis was used to identify patterns in the data. Initial coding was done by using “in-vivo” codes (i.e. applying codes to terms used by participants). This informed the direction of the next stage of analysis, which focused on identifying categories that synthesized the codes and data across transcripts. In this stage, broad themes and sub-themes were developed.

**Findings:**

Six themes were constructed to reflect the guiding principles of UTGSS staff. This includes four central themes: 1) Offering hope; 2) Building respectful relationships; 3) Providing consistent support; 4) Meeting people where they are at. Two connected themes are also reported: 1) *Relying on shared experience,* which participants reported serves as the foundation for practicing these guiding principles and 2) *Bridging connections to services*, which reflects the outcome of practicing these guiding principles.

**Conclusion:**

The principles identified in this study can be used by UTGSS staff as a guide for checking-in on progress with clients and may be considered as a model for reflection on practice by staff providing similar peer-led services. These principles should not be applied in a prescriptive way, as relationship building is at the centre of peer support, and different applications will be required depending on clients’ goals and the range of supports available within their community.

## Background

People who have been incarcerated face a number of barriers transitioning from prison back to community [1–3]. These are driven in part by the significant interruptions to community, health system, family, and peer networks of support posed by periods of incarceration [3, 4]. Upon return to community, the most immediate needs facing this population are income and social support to obtain basic needs, including access to food, clothing, and safe and stable housing [5, 6]. Alongside these immediate basic needs, are significant service needs to address the disproportionately high rates of chronic physical health problems faced by people who have been incarcerated, often coupled with the need for health system support to address mental health and or substance use needs [7, 8].

People who have been incarcerated face social and structural stigma when seeking health and social services. For example, negative and stigmatizing beliefs about people with histories of incarceration and/or substance use and mental health needs can create barriers to securing employment and accessing care and support in social assistance, criminal-legal, and health care settings [3, 9–11]. These experiences of stigma are known to be further compounded for people with particular identities, including Indigenous ancestry, who are overrepresented in the criminal legal system in Canada. Anti-Indigenous racism has been identified as systemic in Canadian health and carceral systems and system-level efforts are required to transform health care culture, policies and practices to move toward health equity and reconciliation [12]. This systemic stigma discourages health and social service engagement, leaving people unsupported and at further risk of adverse outcomes following release, such as unmet housing and financial needs and limited opportunities for employment, return to incarceration, and significant preventable morbidity and mortality [13–15]. As such, eliminating barriers to services access is critical to promoting positive post-release outcomes.

There is a growing body of evidence demonstrating the effectiveness of peer-led services in supporting community reintegration, helping people to navigate the many complex barriers and challenges faced in this period of transition [16–19]. For example, a pilot randomized controlled trial in the United States tested a peer-led support intervention which provided social, emotional, and logistical support to people released from prison to promote health care engagement. People who received the intervention were significantly more likely to access substance use treatment and mental health services, to live in permanent housing, and had a lower recidivism rate than people who did not have access to the peer support services [4]. The evidence supporting these programs builds on decades of evidence from peer-led services in other settings, for example, among people living with HIV, people who use substances and access harm reduction services, and people with chronic diseases such as diabetes, whereby peer support builds a sense of trust which can support readiness to engage in services [20–24].

Despite growing evidence, there remains a paucity of peer-led programs for people released from prison. This has become particularly evident in the context of COVID-19, where interruptions to health and social services have most affected people already facing the greatest inequities, including people who have been incarcerated [25–27]. Health system and policy decision-makers in British Columbia (Canada’s westernmost and third most populous province) are looking to the potential of peer-led programs as an intervention to address these inequities, and minimize the risk of pressing public health threats, such as illicit drug toxicity (overdose) death, which continue to disproportionately affect people released from prison [28–30].

In BC, Unlocking the Gates Services Society (UTGSS) (a not-for profit organization) has been operating a peer-led mentorship program since 2011, offering release planning and community reintegration support for people released from prison. UTGSS is not a direct service provider, but instead is a peer-led organization, whose peer mentors work to connect people released from prison to services in the community, in a client centered way. This includes ensuring access to basic human needs following release, including food, clothing, and shelter. UTGSS has been able to meet significant need, reaching more than 1000 people in 2022 alone.

This study aims to document the guiding principle of a peer-led service for people released from BC correctional centres (herein referred to as “prisons”), from the perspective of peer mentors. The findings can be used to inform the expansion of an evidence-based and often overlooked approach to reducing recidivism and promoting well-being in people released from prison and may have broader application to peer-led services in other populations who face social marginalization, including people living with mental illness and or people who use illegal substances.

## Methods

### Study design 

Qualitative research can best be understood when considering the ontological approach guiding the inquiry. In the present study, the objective was to develop internal documentation of UTGSS principles, practiced by UTGSS Staff in their work connecting clients to services post-release. These principles can best be understood as efforts to understand the questions, assumptions, beliefs, and biases researchers bring to their inquiry. The first author (HP), who led data collection and analysis engaged in memoing regarding reflexivity to reflect on how her position, prior experiences, assumptions, and beliefs might have influence on the results [31]. The first author was an outsider, with no lived experience of incarceration, and approached the research question with a level of naivete not held by other team members, including the UTGSS Program Manager (PY) and Executive Director (MK). The research team, and in particular the first author who led data collection acknowledged her interactions with participants as an inherent part of the research process, where findings are derived from co-construction of knowledge. This is in line with constructivist epistemology, whereby it is understood that research findings are inevitably a construction of our own understanding of the world rather than a purely objective perception of reality [32, 33].

### Study sample and setting

Participants of the present study are UTGSS staff. UTGSS is a peer-led organization that supports people leaving provincial and federal prisons across British Columbia. Importantly, contact to engage in release planning is initiated while people are still incarcerated. UTGSS provide immediate practical assistance (e.g., meeting people at the gate and providing transportation when they are released; support with acquiring clothing, housing), emotional support (e.g., accompanying clients to medical appointments and court proceedings) and facilitate clients in attaining longer term personal goals (e.g., employment) and other needs (e.g., access health and social services) post-release.

### Data collection

The UTGSS Executive Director (MK) and Program Manager (PY) (co-authors) supported recruitment of participants via their network of staff to take part in interviews and/or focus groups. The interview guide was developed in collaboration with MK and PY and included questions about staff motivations for engaging in the work, day-to-day activities, and the key values and principles used to guide their work with UTGSS clients. The interview was trialled with ED and Program Manager before bringing it to the rest of the UTGSS staff, and thus MK and PY served as study participants, in addition to serving as co-authors. As UTGSS staff with significant institutional knowledge, MK and PY have critical insights to offer as to the guiding principles practiced by staff.

Data were collected by the first author (HP) using a mix of focus groups (*N* = 10; 2 groups with 5 participants each) and one-on-one interviews (*N* = 5) including a total of 13 people, representing all UTGSS staff at the time of the study (Two interviews were conducted with people who also participated in the focus groups) (See Table 1). One-on-one interviews were offered to collect data from people who were unable to attend the focus group, who preferred a one-on-one conversation to a group discussion, or in cases where points raised in the focus groups needed to be clarified or expanded on. Participants received the consent form and information about the study prior to data collection. All participants provided informed verbal consent prior to beginning the focus group/interview. All data were collected virtually on zoom, in accordance with COVID-19 protocols. Data collection began with an overview of the project and its purpose, followed by reviewing a safety protocol to promote a safe and respectful discussion (in focus groups only). Participants were invited to make additions or modifications to the safety protocol. One-on-one interviews lasted between 40–60 min and focus groups lasted between 90–120 min (including consent, and review of meeting procedures and safety protocol). The interviews and focus groups were audio-recorded and transcribed. At the stage of transcription, all identifiers were removed and participants were assigned a pseudonym. An amendment was entered with the University of British Columbia Behavioural Research Ethics Board (UBC-REB), who approved the presentation of names rather than pseudonyms for MK and PY, at their request. Participants were provided $50 cash honorarium for their time. Research Ethics approval was received from the UBC- Behavioural Research Ethics Board (BREB) (#H21-03046) and the study procedures, including approach to recruitment, data collection, compensation, interpretation, and knowledge translation have all been approved in accordance with BREB policies.

**Table 1:** Demographic characteristics of study participants

Characteristic	N(%) *N* = 13
**Self-identified gender**	
Woman	6 (46.2)
Man	7 (53.8)
**Self-identified Indigenous ancestry**	
Yes	6 (46.2)
No	7 (53.8)
**Age**	
*Categories*	
30–39	2(15.4)
40–49	5(38.4)
50–59	5(38.4)
60 +	1 (7.7)
*Mean Age*	48.77 ± 8.27
**Region of province providing services**	
Fraser	5(38.4)
Vancouver Island	2(15.4)
Interior	3(23.1)
Northern	3(23.1)
**Years working with UTGSS**	
*Categories*	
≤ 1 year	4 (30.8)
2–4 years	4 (30.8)
5–10 years	3 (23.1)
≥ 10 years	2 (15.3)
*Mean number of years*	4.69 ± 4.91

*N* = 13 unique people; Northern and Interior have lower density, more rural regions, Fraser and Island more density

### Data analysis

An inductive thematic analysis was used to identify patterns in the data. In line with Braun and Clarke’s approach to thematic analysis, transcripts were first read to gain familiarity with the data prior to beginning analysis [34]. Initial coding was done by using “in-vivo” codes (i.e. derived from the data itself, applying codes to terms used by participants) and were discussed amongst co-authors (HP, MK, PY, AKS) which informed the direction of the next stage of analysis, which focused on identifying categories that synthesized the codes and data across transcripts. In this stage, the broad themes and sub-themes were developed, which each reflect actions and words used by participants (e.g. offering hope, meeting people where they are at). This was a data-driven approach, and relied on discussions between members of the team, supported by visual representations of the data (i.e. with subthemes mapped visually and connections between them drawn and documented during team discussion). This process supported the team to collaboratively to review and revise themes, understand overlaps, distinctions, and relationships between sub-themes which supported decision making regarding moving toward determination of final themes.

**Fig. 1:**
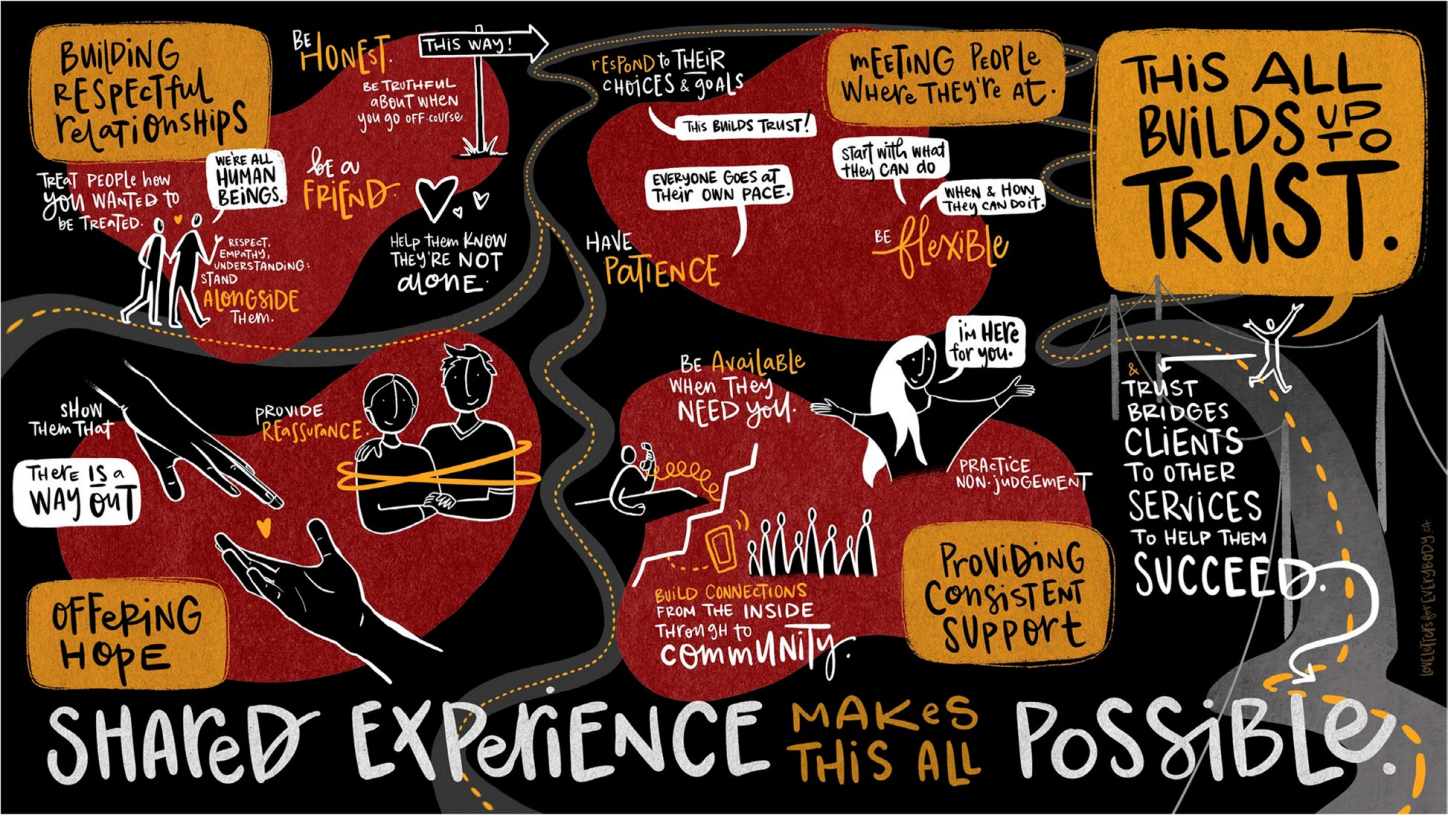
Graphic illustration of UTGSS staff guiding principles

These themes were then brought to a UTGSS staff team meeting, for discussion. The meeting was attended by a graphic illustrator who documented the themes (See Fig. 1). Minor changes were made following the meeting to refine codes, but no changes were made to major themes or guiding principles. From the discussion, it was agreed upon that the two themes, “relying on shared experience” and “bridging connections to services”, should be incorporated into the manuscript to provide important context for the guiding principles. As such, the data is organized around six themes, four of which reflect guiding principles, as well as “relying on shared experience,” reflecting the foundation for the guiding principles and “bridging connections to services,” reflecting the outcome of practicing these principles (See Table 2).

**Table 2:** UTGSS staff guiding principles: Themes and Sub-themes

Theme	Sub-themes
**Relying on shared experience**	
**Offering hope**	Showing clients a way out of the revolving door of prison
	Providing reassurance
**Building respectful relationships**	Treating clients how you want to be treated;
	Being honest
	Being a friend
**Providing consistent support**	Building connections from inside prison through to long-term community reintegration
	Being available when needed
	Practicing non-judgement
**Meeting people where they are at**	Being flexible
	Having patience
	Responding to clients’ choices and goals
**Bridging connections to services**	Offering an extra push
	Providing credibility to services

## Findings

Data were collected from 13 UTGSS staff (Self-identified gender: *N* = 7 men; *N* = 6 women; Self-identified Indigenous ancestry: *N* = 6; Average age: (Mean ± Standard deviation (SD) 48.77 ± 8.27)). On average participants had worked with UTGSS for (Mean ± SD: 4.69 ± 4.91) years, and worked in each of health regions in BC where provincial prisons are located: 1) Fraser Health (*N* = 5); 2) Island Health (*N* = 2); 3) Interior Health (*N* = 3); 4) Northern Health (*N* = 3). Participant demographic information is listed in Table 1. From the data, there were six themes constructed to reflect the guiding principles of UTGSS staff. This includes four central themes: 1) Offering hope; 2) Building respectful relationships; 3) Providing consistent support; 4) Meeting people where they are at. The sub-themes within each of these four guiding principles reflect the ways in which these mechanisms are put into action, providing examples of the actions taken (or the “how”) to carry these principles out in practice.

Two connected themes arose as adjacent to answering the main research question: 1) *Relying on shared experience,* which participants reported serves as the foundation for practicing these guiding principles and 2) *Bridging connections to services*, which reflects the outcome of practicing these guiding principles. The supporting theme, “*relying on shared experience*” will be introduced and described first to provide context around the work of UTGSS staff. The second supporting theme “*bridging connections to services*”, will be outlined following description of the four principles, as this theme represents the outcome of staff’s work practicing these principles.

### Relying on shared experience

Participants described a shared understanding of clients, through language, challenges, and feelings that they experienced in their transition back to community. They described that the experience of incarceration and return to community cannot be taught, and a true understanding can only be brought by someone who had also lived through the same experience. For example, Simon described:

*“It’s like if you’re a mechanic and you go hang out with a bunch of other mechanics, everybody knows what you’re talking about. When you’re talking about the “thingy majiggy” that’s under the other thing. Whereas somebody else that maybe has read about it, they would know but they wouldn’t know the experience of it. So I think that’s where, where the like peer programs are most important. It’s not the book knowledge and all that. You know, the resource knowledge and stuff. It’s the shared understanding of the experience.”*

-Simon, Peer Mentor

Participants noted a vulnerability in clients during the period of return to community. Reciprocating the same vulnerability by openly sharing personal experiences was an important approach to building connections with clients, relying on shared, collective experiences. For example, Kent described that in opening up, and sharing his own experience with clients early in their relationships, he was able to develop stronger communication and a sense of trust:

*“The people that I have met, when you let them know that you’ve been down somewhat of the same road, people open up to you much more easily. They have a feeling that some of our realities have been the same, and that opens up a path to communication and trust. So, I think that’s, what’s important for people who are just being let out is that they are able to be in touch with peer mentors, like us from Unlocking the Gates”*

-Kent, Peer Mentor

The shared experience motivated staff to support UTGSS clients, with the aim of making the process of navigating service access easier than it had been for themselves. Staff frequently reflected on their own struggles faced during their transition back to community and were able to use these experiences to identify important points of intervention, where they could target supports to avoid similar negative experiences for their clients. For example, Yelena said:

*“I want to give people the extra hand because I’ve been there and I know how the system can be. All these things [services] are available to us, but they make it hard for us to access them, especially if you’re coming from the street. And I know because I dug myself up from the street.*

*I know what it’s like.”*

-Yelena Peer Mentor

### Offering hope

The focus on shared experience sets the foundation for the work that UTGSS staff do, including their work focused on the first guiding principle of “*Offering hope*”. Participants described the time immediately after release from prison as overwhelming and stressful, filled with tremendous, ranging emotions and hurdles to overcome. This period was characterized by the need for clients to receive compassion, empathy, and encouragement regarding the next steps in their lives. Offering hope was a foundational core principle practiced by the UTG staff. Discussions of offering hope were focused around: 1) showing clients a way out of the revolving door of prison; 2) providing reassurance.

Observing successes of UTG peer mentors, specifically, stability in terms of housing and health, was noted to offer clients hope for possibilities regarding their own future. Mo, UTGSS Executive Director described the lack of connection and support people are met with when they are released, and the gap that UTGSS peer mentors can fill to offer hope to clients by “*showing them a way out of the revolving door of prison”:*

*“A lot of times people who are going through a lot, they, they don’t feel loved. They’ve lost their families. They’ve lost their friends, they’ve lost employment, they don’t know how to use a computer, they’ve never used a cell phone. They just don’t feel a part of the world they are coming out into, and sometimes they just want to sabotage themselves to go back to the safety of what they know. So, it’s just a matter of showing that there is a way out.*

*Just by seeing somebody who has found a way out and I think that’s a big thing.”*

-Mo, UTGSS Executive Director

Given these challenges to community reintegration, peer mentors described the process of re-engaging in community as non-linear, with clients often being met with setbacks. Having consistent contact with UTGSS staff, to provide reassurance through these difficult times was described as critical to supporting clients in remaining hopeful about the future. Neil described that some clients often need this sense of encouragement and, when it comes from someone who has overcome the same challenges, it is all the more meaningful:

*“A lot of times these guys just want somebody to say, hey, you know, it’s not so bad. You’re okay, too. Everybody needs a little bit of reassurance and understanding sometimes that, you know, everybody makes mistakes. I’ve made them. Just to say, hey, it’s not so bad out here, and it takes a while. Sometimes it takes a bit to get things going and to get started, but it’ll come to you just like it came to me.”*

-Neil, Peer Mentor

### Building respectful relationships

Another core principle described by UTGSS staff was building respectful relationships. Three approaches were consistently discussed in this regard: 1) treating clients how you want to be treated; 2) being honest; and 3) being a friend.

Foundational to building respect among UTGSS staff was treating clients how they wanted to be treated. This experience required reflection on their own feelings and experiences at the time of release, including recalling encounters with health and social service providers, whether negative or positive. For example, one participant reflected on their own experiences in health care following release, feeling unsafe, and unable to discuss their drug use with their doctor due to feeling they were being judged about ongoing use. Health care visits were recognized as interactions with vast power imbalances between patients and providers and having a peer accompany clients to their visits was seen as a way of minimizing this imbalance. In these discussions, participants emphasized the stigma and discrimination facing people released from prison, and that their interactions were intended to meet each person with respect, regardless of their current situation. Ellen elaborated:

*“Treat them how you want to be treated that is my main policy to live by. I want to treat people the exact same way I would want to be treated when I got out of jail or when I went into a courtroom. If I was wearing ripped, baggy jeans with holes in them, I would want to be treated the same way that somebody walking in with a lawyer’s briefcase is treated. That is my big value I remind people of over, and over again that we’re all still human beings and that we all need to be treated that way.”*

-Ellen, Peer Mentor

Building respectful relationships was also accomplished by practicing an approach based on honesty. UTGSS staff described that in their own experience following release, having people who were honest with them about areas that they may need to improve was something they respected in their mentors, and was critical to their progress. This involved UTGSS staff mirroring behaviors, such as being accountable to clients in terms of consistently being honest with them about their perceptions of their plans, and how they may or may not be best serving their goals. Simon explained how being honest with clients allowed them to recognize areas in which they may need to take action or change their approach in order to stick to the plans for community reintegration they had made following release:

*“Throughout my life, I wish people would’ve been honest with me and say I was messing up when I was messing up. You know, it might have been helpful. Because we have that shared experience and we’ve been there, and we can relate. When things are starting to go sideways, we can kind of recognize that. I think they really, respect being straight up and being able to kind of call them on their bull shit sometimes. You know, in a kind way, in a good way just to be able to say hey, maybe, you might be trying to leave yourself a back door. They might have the best of plans while they’re inside corrections, but once they get out in the community and they start getting overwhelmed, you know, just being able to call them on it and help them, help them recognize when things are going sideways.”*

-Simon, Peer Mentor

Following discussions about honesty, was an emphasis on friendship. UTGSS staff explained how their role did not require stringent boundaries and detached professionalism. They compared their work to other professional roles, such as nurses or social workers, and described their goals as being different: they focused on building friendships and demonstrating a genuine sense of care for the wellbeing of the clients. The staff recognized that many of the events that clients need to face following release can be extremely difficult to navigate alone and are experiences that require emotional support. For example, Yelena described going to court as an overwhelming experience that people may avoid if they do not feel equipped to navigate the emotions and stress. She outlined how supporting one client through the court process required her to first, and foremost, show up as a friend:

*“I just try to be there for them, and be a friend, someone who cares for them. They could feel that, you know, and I chat with them, and I make them feel comfortable. Even just arriving to an appointment or holding their hand in court. Sometimes people want that, it’s silly, but it’s true. We all want support; we don’t want to go through that [going to court] alone.”*

-Yelena, Peer Mentor

### Providing consistent support

Another core guiding principle that was identified in the analysis, was providing consistent support. This was accomplished by: 1) building connections from inside prison through to long-term community reintegration; 2) being available when needed; and 3) practicing non-judgement.

UTGSS initiates client support and services while people are still incarcerated. This allows for the development of release plans to ensure that supports are made available in advance of the person’s return to the community. For example, housing and treatment centre admission can be arranged so that clients have a place to go immediately upon release. UTGSS staff explained that this engagement before release is critical to building trust, rapport, and to preparing clients for success following release. The period of transition was described as one of uncertainty, and staff can play a role in ensuring clients are in a position to be released into a context that is most conducive for them to meet their goals. For example, Pam described that she works to understand clients’ needs and goals prior to release, and discussed a case where she was able to get to know a client’s preference for accessing housing in proximity to his family, which she was only able to arrange prior to release by engaging in contact with the client while he was still incarcerated:

*“I just had a call with someone who is in corrections and was talking about how they’ve reconnected with their family since they have been back in and they hadn’t talked to them in three years and how excited they were about reconnecting with family and their kids. So, I am just being that person who can be encouraging and try to help them with a plan that will, place them closer to their family in a community when they are released.”*

-Pam, UTGSS Program Manager

Consistency of support relies on connections from inside, through to community, and regular and reliable contact between clients and staff. This requires staff to be flexible and available, responding to clients’ requests when they are made. Many staff described the ways in which services operating only on regular business hours, or with waitlists posed dramatic barriers to clients’ ability to reintegrate into the community. UTGSS staff work, to the best of their ability, to be available when clients need them. For example, Brian outlined his commitment to answering phone calls from clients, regardless of the time of day:

*“I try to take the opportunity to answer the phone, if the individual calls, that’s a big one for me. I would get so frustrated when, I would try to access resources and I would call a number and there wouldn’t be a voice on the end of the line and no call back or, or anything. So, it’s like just a typical day for me is, is making sure that I’m available and accessible to the best of my ability and try and figure out a solution to help those in need.”*

-Brian, Peer Mentor

Central to continued and consistent support, were discussions about providing non-judgemental support, and continuing to show up, regardless of the “progress” clients made. Staff described how during their own transition to community, they had faced supports that were conditional. For example, they may have been denied services, or had their access to a service discontinued if they were not abstinent from drugs. Staff described that these sorts of restrictions push people further from services and make re-engagement more challenging. UTGSS staff emphasized that consistent support meant that they were present to respond to the requests, needs, and goals of the clients, without judgement. Staff outlined the importance of still being there to provide support when clients were ready. For example, Simon outlined a situation where a client did not follow through on his release plan. Instead of declining to support, Simon followed the client’s wishes and assured them they could get back in touch if they needed assistance in the future:

*“I am going to drop them [client] off, you know, where they want to go. All I can do is give them some resources, give them my card and say if you get stuck give me a shout. If you get stuck or if you go back to jail, call me right. I’ll drive you up [from jail to home] again.*

*I have no problem doing that.”*

-Simon, Peer Mentor

### Meeting people where they are at

The last guiding principle that was identified in the analysis, was “*meeting people where they are at*”. This was accomplished by: 1) being flexible; 2) having patience; 3) responding to their choices and goals.

Participants outlined that no day was the same in their work with UTGSS. Each day, their work was planned around the needs of the clients. Sometimes, this meant following the initial plan, while other times, their plans were adjusted to respond to the priorities of the clients. Participants described that when clients are released, they all have different needs and priorities, and sometimes their priorities might change between the time they developed their initial release plans, and the day of release. Staff described seeing clients holistically, with a range of health and social needs. They work to prioritize those needs which clients identify as most pressing and most important. Ellen described that flexibility was critical in her role. She shared that her responsibility in her role is not to accomplish a pre-determined checklist, but to be flexible and plan her day around the needs the client presents to her when she meets them:

*“You can’t plan your day or to be the same all week long and be set on a schedule because it doesn’t work that way. We have to be really flexible to be able to do certain things and meet the clients where they’re at. Whether we are getting them clothing, whether they’re cashing a check, taking them to probation, or to court, or signing them up for Hepatitis C treatment, each day is different. We have to be really flexible to be able to meet them at where they are and to be able to get all their needs taken care of.”*

-Ellen, Peer Mentor

Staff also described the need to practice patience in their work with clients. Clients’ progress toward their goals was not always linear and took time. Brian described that clients will develop their own goals and access supports in their own time. It is the role of the peer support worker to encourage them, and step in to provide connections to resources that are in line with the goals of the client, when the client is ready for them. Brian stated:

*“It can be frustrating because you could lead someone to water, but they’re not always willing to drink. I try not to write anybody off because I’ve been there too. And I know that, at some point, my hope is that, they would actually put one foot in front of the other and access those supports and make those calls. [When I was released] I had somebody come alongside me and, and was patient, compassionate, empathetic, and would encourage me, would assist me in coming up with my own solutions and not just coming up with a plan of what they thought was best for me. They actually listened to some of the concerns that I had and actually came alongside me, just to support and encourage me like that.”*

-Brian, Peer Mentor

Following the focus on flexibility, and patience, was the focus on being responsive to the clients’ goals and choices and acknowledging that ultimately these choices were not up to the staff, but to the client. Staff described sticking by the clients, regardless of whether the clients’ goals matched those that the staff envisioned as being best for them. Even where initial plans were not followed, staff took a strengths-based approach, refocusing to find ways to support clients in whatever path they chose, recognizing the benefits of consistent, reliable support. The strengths based approach draws from the practice of social work, and assesses the strengths and resources that are present for an individual in their community, and seeks to build on these strengths or resources to resolve challenges or difficulties [35]. Ultimately this practice is reflective of a harm reduction approach, whereby services and supports are offered to minimize harm for the client, under whatever circumstances they currently face. Staff practiced this approach skilfully, and respectfully. For example, Pam outlined that when clients denied wanting to access specific services she was suggesting, she would instead reframe her focus to asking the client how she could best serve them, asking them what they needed and what would be most helpful to them in that moment:

*“I think it’s all about meeting people where they’re at. Like if someone decides I don’t want to go to the shelter, you just have to try to support them in that decision. You can try to convince them, maybe, you know, it’d be a better idea if you went to the shelter so you have housing and support. But if they don’t want to go to the shelter, ultimately, it’s their decision and you just support them in that decision. You can ask how can I help you? Can I help get you a camping package? Can I help get you some food or, you know, blankets? You just support them in whatever decision they choose to make, because ultimately it is their choice.”*

Pam, UTGSS Program Manager

### Bridging connections to services

Ultimately the most consistent positive outcome of the work that UTGSS staff accomplished by practicing their guiding principles was building a sense of trust that allowed them to help clients bridge connections to services. This was accomplished by: 1) offering an extra push; and 2) giving credibility to services.

Staff noted that many clients wanted to connect with services but did not know where to begin, facing critical barriers to accessing them (e.g. lack of transportation to appointments), or were overwhelmed or uncomfortable accessing them independently. This led to people falling through the cracks, either missing medical appointments, or court dates, leading to longer-term challenges. Yelena described how she supported clients in navigating the barriers to accessing services:

*“So I’m supporting with rides to get to court and to get to probation. Half the people I’ve talked to said, you know, they don’t even want to check in [with court]. They’re not going to check in or they are forgetful and need a ride, and it becomes such a hassle in their lives. Then they end up breaching and they ended up going back to jail and it’s just like a whirlwind with them.”*

Staff also described how clients just needed a little bit of extra encouragement or support to connect with services. For example, Yelena described a situation where she provided encouragement for a client to go to court:

*“Today I went and took a lady to court and then I saw another lady outside of the probation office that hasn’t checked in in a long time, but she was right there. I asked “if I walk in with you, will you go?” She said, yes. So, I took her in and got her checked in I’m glad I did that with her because we wouldn’t want her breached and then in jail. People just don’t want to walk into that kind of building by themselves. I just think she would’ve called eventually, so it’s like, we’re kind of pushing them to help themselves.”*

-Yelena, Peer Mentor

Staff described how having been incarcerated creates a general sense of distrust in the health and justice systems, due to traumatic experiences, including denial of needed services. UTGSS staff who have accessed these services themselves, or who have previously supported other clients to access them are able to lend a sense of credibility to services. For example, Simon described that he was able to make meaningful referrals to his clients that they otherwise would not have followed up on:

*“When I suggest a professional for them to go visit, I call it like a [prison] yard. It’s like when you meet somebody in the [prison] yard inside prison and you say “they are alright”. Then people will tend to, to say, okay, well he is alright. So, I think we have that ability when it’s one of our own, basically saying, you know, go see this lady she’s going to help you. We give them a little bit of credibility because we come from the same world and we speak the same language, and then we can get them into services easier. It is quite helpful to have somebody that knows. That has lived it.”*

-Simon, Peer Mentor

## Discussion

It is important first and foremost to acknowledge the scope and limitations of this paper. The objective of the paper was to outline the guiding principles of the work of UTGSS staff. We therefore represent only the perspectives of staff, and not clients who may have a wide range of experiences and perceptions of the peer mentorship offered by UTGSS. The client perspective is critical to understanding the impact of UTGSS’s peer mentorship. Our team is leading ongoing studies with a focus on collecting client perspectives. These data will be made available in upcoming publications to provide a more holistic perspective on the impact and reach of the services and supports offered by UTGSS.

The objective of this study was to identify and document the principles guiding the work of UTGSS staff as they support people released from prison in their transitions to community. In other settings, such as in the provision of mental health services, the documentation of principles guiding peer-led approaches has been described as a critical step to support further development and evaluation of peer-led programs as they become more consistently and commonly incorporated into “mainstream health services” [36]. As such, in the context of growing attention to the role of peer-led services in bridging connections to health, this study provides foundational knowledge regarding principles that can inform the evaluation of peer-led services and advocacy for expansion of these services, for people released from prison, but also for other groups (e.g. people with HIV, HCV, mental health or substance use needs) for whom peer-led services have been shown to play a role in bridging connections to care [37–39].

In this study, we identified principles that guide the peer mentorship work of UTGSS staff, who represent the only non-profit agency providing direct and continuous services to people released from prison in BC from release planning through to community reintegration. We found that this work was made possible through the shared experience these staff held, which facilitated trust, and resulted in connections to services, which otherwise would not have been possible. Shared experience has been identified as a foundation of peer-led work in other populations, including people experiencing homelessness, and people living with mental illness [40–42].

Prior studies have defined shared experience as providing an innate ability to generate trust, empathy and open communication that was only possible because of the credibility peer mentors gained by sharing with clients that they had been in their situations before [41].

The first guiding principle that was made possible through this shared experience, was “offering hope”. Prior studies have outlined the concept of hope relative to peer work, by discussing the positive impact peer work can have on identity transformation, for people who have often lived with shame associated with the identity of having been incarcerated that follows them upon release to community [43]. The concept of hope has been described as reciprocal, where it provides an opportunity for peer mentors to reflect on their process and positive work and define their sense of self around these positive aspects of their lives and promote the same in their clients [44]. UTGSS staff outlined that this sense of hope provided clients with reassurance and support in taking actions needed to support their community reintegration, consistent with the literature on self-efficacy and hope in promoting health behaviour change [45–47]. This is consistent with the growing literature on the role of peer mentorship in the carceral context, where it has been acknowledged to support people to realize their personal strengths and imagine new identities [48].

Participants of the present study also outlined the critical focus on building respectful relationships as a foundation of their work. One central component of relationship building was *“being a friend”.* Participants outlined how they were able to fill a gap that other professional service providers are unable to, as these connections are not commonplace or acceptable for staff working in existing health, social, and criminal legal system services. Given the interruptions to social networks posed by incarceration, many clients did not have access to a network of friends or family who could support them to navigate the systemic hurdles required as part of the process of reintegration (e.g., probation appointments, court dates, etc.).

The study findings confirm the critical importance of building respectful relationships to promote health and social equity for people during community reintegration following incarceration. While health and justice professionals also know the importance of relationships for improving equitable care, the systems within which they work deprioritize relationships in favor of efficiency and client processing in inadequately resourced and siloed systems of care. Peer-led programming and engagement is critical for addressing the impact of power relations and institutional policy impacts faced by people released from prison in their transition to community [36]. The principles described here are foundational to the everyday work of reducing inequities in accessing care and when put into practice, can lead to improved reintegration experiences and outcomes.

Prior studies of peer-led programs have identified that once program expansion occurs to more standardized organizational environments, the guiding principles and values of peer-led organizations can become more and more difficult to maintain [49]. This does not preclude health and justice systems from establishing or expanding peer-led services or incorporating peer experiential workers into existing programs, as there are undoubtedly benefits in these models, as demonstrated by prior studies [4]. This does however highlight a gap filled by UTGSS, who provide a peer-led service, where the direction of service provision remains independent of the directives of health and justice organizations. This is critical to allowing UTGSS staff to maintain and practice the principles outlined, such as *“providing consistent support*” without logistical (e.g. working hours) and policy (e.g. service mandates) barriers to service provision that might be imposed when peer-led services are incorporated into more mainstream health services [36].

UTGSS staff support clients across the province, while regional health organizations may only have mandates to provide services within their health region. These mandates can translate to clients experiencing gaps in care. For example, if the region of the province where the client is released from is not where their residence is, responsibility for care often needs to be transferred between health authority services in two different regions. Gaps in care have been well documented when handovers such as this occur between different providers or services [50]. UTGSS staff provide navigation and support that is across regional health boundaries and therefore assist in providing a ‘warm handover’ between services in different regions and enhance continuity of care for patients. Both forms of peer support, those provided within mainstream health or justice services, and those provided by peer-led organisations such as UTGSS, are vitally important as they each have distinct benefits. The UTGSS role can be viewed as an adjunct or support for mainstream health services, even where peers are already integrated into programs, and both may further enhance each other’s efforts through collaboration and coordination. Nevertheless, there remains a need for further recognition and resources for peer-led organizations to support their operations and long-term sustainability. This has been well-documented when considering peer-led responses to the ongoing drug toxicity crisis. For example, staff often derive meaning and fulfillment from their work, but burnout and stress is also consistently highlighted [51–53]. Recognition of the work, need for organizational support, ongoing skills training, and development have been highlighted as critical to supporting peer workers in overdose response settings, all of which have application to peer work in broader settings, including among UTGSS staff.

Ultimately, the outcome of the principles practiced by UTGSS staff was better connection to health services among a population who has traditionally faced many barriers to health service engagement [1–3]. Discussions about engaging or re-engaging people who have been incarcerated in health care must acknowledge the founded distrust this population holds toward the health and justice systems. In Canada, Indigenous peoples are known to disproportionately face criminal charges [54], and while Indigenous peoples make up only approximately 6% of the population in BC, they represent 35% of people incarcerated in provincial prisons [55]. Indigenous peoples in Canada have been impacted by the traumas and injustices of colonialism, including residential schools, the 60 s scoop, and the foster care system which has produced justified mistrust in government agencies [56]. This mistrust rightly follows people to community and remains in place relative to considerations for engaging in health care settings, which are known to be spaces where Indigenous peoples face systemic racism [12]. As such, UTGSS’s independence from the health system is critical to building trust. This independence or separation from the health and justice systems has been identified as an important part of operations among peer-led services for other populations. For example, in mental health settings, peer support has been defined as separate from and even in resistance to traditional mental health services where care is medicalized and relies on power structures of expert to patient, which are very different from more horizontal peer-to-peer relationships. As such, there is a critical benefit in terms of potential for rebuilding trust that results from UTGSS’s independence from government agencies. This independence is important to acknowledge and to maintain where the goal is to engage people in care who have justified mistrust in the system.

While this manuscript has primarily focused on outlining a positive approach to the work, with guiding principles that seek to ensure clients receive the best service, it is important to also acknowledge that UTGSS staff are faced with a number of constraints and challenges in their roles. First, it is important to acknowledge that UTGSS staff are operating in British Columbia, a Canadian province that has been experiencing an ongoing public health emergency of illicit drug toxicity (overdose) deaths since 2016, which has worsened in the context of COVID-19 [57, 58]. Overdose is the leading cause of death for people aged 10–59 in the province, and people released from prison in BC face a disproportionate risk of drug toxicity death [28]. UTGSS staff hold a strong sense of responsibility in seeking to connect clients to care and seek to offer the best level of service to clients Often however, social services are limited, and staff may be unable to provide clients with direct referrals to housing, or treatment services and programs when they need them which takes a direct toll on staff morale and well-being. Nevertheless, UTGSS staff continue to show up and make themselves available to clients, which has supported their growing credibility in the community, and with related organizations (e.g. organizations providing correctional leadership, probation officers, etc.).

This paper contributes to the literature on peer-led services by outlining guiding principles that could serve as a foundation for other peer-led work, to foster enhanced care across diverse populations. UTGSS supports community integration and access to health and social services among people being released from prisons in British Columbia. The principles outlined here have relevance to people with overlapping or similar mental health and substance use service needs. Practically, these principles can be used by UTGSS as a guide for checking in on progress with clients and may be considered as a model for reflection by staff providing similar peer-led services, to reflect on the extent to which they have been able to practice each principle in engagement with each of the clients they serve. This can be done individually, or in group settings, where UTGSS peer mentors suggested this sort of reflection could be an insightful exercise to review as part of regular team meetings. This should not be applied in a prescriptive way, recalling that relationship building is at the centre of peer support, and different applications will be required depending on clients’ goals and range of supports available within their communities.

## Data Availability

The transcripts analysed during the current study are not publicly available but may be available from the corresponding author on reasonable request.

## Abbreviations

BC: British Columbia
HCV: Hepatitis C Virus
HIV: Human Immunodeficiency Virus
UTGSS: Unlocking the Gates Services Society
